# Development of Palladium and Magnetite-Coated Diatomite as a Magnetizable Catalyst for Hydrogenation of Benzophenone

**DOI:** 10.3390/ijms26073157

**Published:** 2025-03-28

**Authors:** Ádám Prekob, Balázs Szeleczki, Zsolt Veréb, Csenge Nagy, László Vanyorek, Ferenc Kristály, Zsolt Fejes

**Affiliations:** 1 Institute of Chemistry, University of Miskolc, Miskolc-Egyetemváros, 3515 Miskolc, Hungary; vereb.zsolt@student.uni-miskolc.hu (Z.V.); nagycsenge.mail@gmail.com (C.N.); laszlo.vanyorek@uni-miskolc.hu (L.V.); zsolt.fejes@uni-miskolc.hu (Z.F.); 2 Higher Education and Industrial Cooperation Centre, University of Miskolc, Miskolc-Egyetemváros, 3515 Miskolc, Hungary; balazs.szeleczki@uni-miskolc.hu; 3 Institute of Mineralogy and Geology, University of Miskolc, Miskolc-Egyetemváros, 3515 Miskolc, Hungary; ferenc.kristaly@uni-miskolc.hu

**Keywords:** diatomaceous earth, magnetizable catalyst, hydrogenation of benzophenone, solvents, characterization

## Abstract

A naturally derived silicate, diatomaceous earth has been endowed with magnetic properties by depositing magnetite nanoparticles on its surface. Palladium crystallites were created on the resulting magnetizable catalyst support. The support provided high specific surface area with high porosity which were ideal for the binding of both the magnetic particles and the palladium. The catalyst was successfully tested in the hydrogenation of benzophenone in three different solvents (methanol, ethanol, and isopropanol). Significant differences in catalytic activity were observed, allowing selective production of benzhydrol (BH) or diphenylmethane (DPM) by a simple solvent change. Beside the excellent selectivity, the featured catalyst also provided an easy and fast method for catalyst recoverability using a simple magnet.

## 1. Introduction

Benzhydrol (BH) is an important organic compound in perfumes and the pharmaceutical industry. For perfumes, it is often used as a fixative. In pharmaceuticals, meanwhile, it is a widely used precursor in antihistamine production [1,2]. Its preparation can be carried out by Grignard reaction, electrocatalysis, photocatalysis, transfer hydrogenation, or direct benzophenone hydrogenation [3,4,5,6,7,8,9]. For mass production, catalytic hydrogenation might be the better choice since it is a well-known and widely used method for continuous chemical production. For this purpose, hydrogenation of benzophenone is a possible way [10,11,12]; on the other hand, it is important to prevent over-hydrogenation, which instead could afford diphenylmethane (DPM). However, diphenylmethane can also be valuable since it is often used for the synthesis of luminogens or diphenylmethyl-potassium, the latter being utilized as a polymerization initiator. Since the separation of BH and DPM is circumstantial, the best approach is to use a catalyst that selectively produces either product depending on the reaction conditions. In terms of catalysts, the most common supports are active carbons, different metal alloys (e.g., Ni–B, Ni–Cu, Ni–Fe), TiO_2_, zeolites, and SiO_2_ [1,11,13,14,15,16].

Diatomaceous earth is a siliceous sedimentary rock with a typical SiO_2_ content of around 90 wt%. Its high thermal stability, specific surface area, and porosity, provided by the uniquely shaped grains that compose the material, render it a great catalyst support. The high porosity makes them an exceptional support by providing many preferred binding sites in terms of energy for different nanoparticles since the particles prefer to bind at sites with high energy (e.g., edges of pores). Furthermore, diatomaceous earth has excellent chemical resistance, low cost, and is 100% of natural origin, which can render the catalyst preparation process environmentally friendly [17,18]. By selecting the appropriate natural deposit, the purity of the diatomaceous earth can be high, which could further enhance its properties.

Diatomaceous earth has already been used as a support for various nanoparticles, mainly transition metals and their oxides, and precious metals (e.g., palladium and platinum). These supported catalysts can be applied for catalytic processes such as oxidation of carbon monoxide, toluene, and ethyl acetate, photocatalytic degradation of dyes, methane reforming, or hydrogenation of vegetable oils [19,20,21,22,23,24]. The main goal of applying these nanoparticles is to enhance the catalytic properties of the noble metals in terms of activity or selectivity. However, it is also possible to introduce new features for a catalyst like magnetic recoverability by forming magnetizable nanoparticles on the support surface.

The common catalysts are usually separated by time consuming and expensive filtering or sedimentation processes after the reaction which can be avoided by using magnetic catalyst carriers (e.g., different iron oxides), since materials with paramagnetic properties can be easily collected and separated from the media using a magnetic field [25,26,27]. Although the magnetic material itself might not function as an efficient catalyst carrier, preparation of paramagnetic nanoparticles on a non-magnetic support (e.g., SiO_2_) as the original carrier is also possible.

Using different solvents is a simple way to further modify the activity of a catalyst and, therefore, the outcome of the reaction. The solvent has a significant influence on the reaction route, reaction rate, and product selectivity due to its individual capability for dissolving hydrogen, thermodynamic interactions with the catalyst and reactants, solvent polarity, hydrogen transfer ability, etc. [28,29]. In case of benzophenone hydrogenation, it is extremely important to keep the reaction under control since the first product (benzhydrol) can be easily over-hydrogenated (to diphenylmethane). Taking these into account, the solvent must be chosen with care.

In this paper, we would like to present a SiO_2_-based catalyst with easy preparation and recoverability that offers high selectivity for benzhydrol or diphenylmethane, depending on the solvent used.

## 2. Results and Discussion

### 2.1. Results of the Characterization

**Figure 1:**
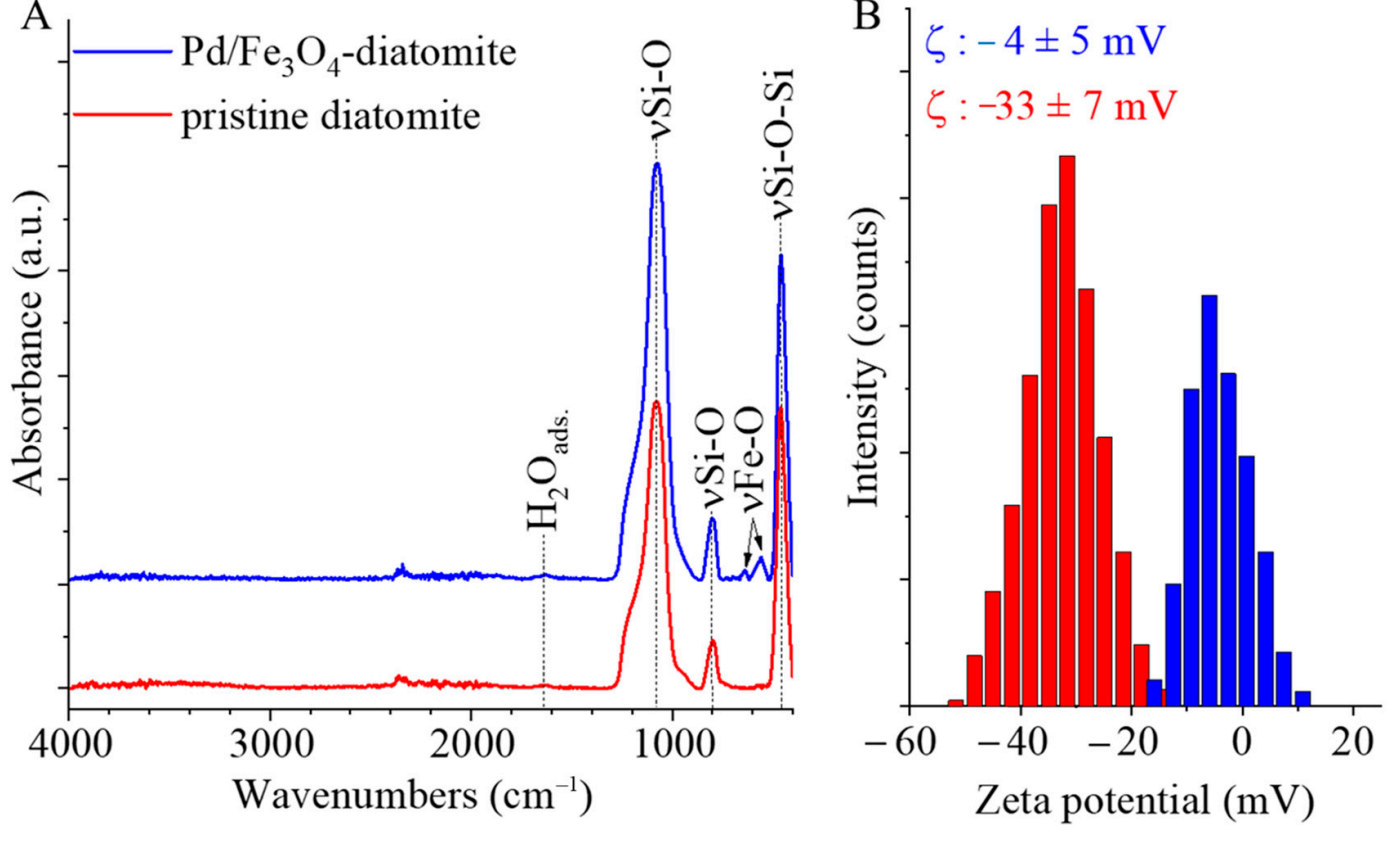
FTIR spectrum (**A**) and zeta potential distribution (**B**) of the pristine (red) and palladium and magnetite-decorated (blue) samples.

Characteristic infrared vibrational peaks of the catalyst were determined by FTIR. The characteristic absorption bands of the Si–O–Si bonds of the pure, dried diatomaceous earth were identified at 1075, 798, and 459 cm^−1^ (Figure 1A) which are attributed to antisymmetric stretching, symmetric stretching, and bending modes, respectively, in diatomite [30,31]. In addition, a small absorption band at 1639 cm^−1^ can be assigned to O–H deformation of adsorbed water molecules [32]. The pristine and the palladium and magnetite decorated diatomite samples were dispersed in distilled water, and their zeta potential values were measured (Figure 1B). In the case of pristine diatomite, the average zeta potential was −33 ± 7 mV, which is sufficiently negative to maintain electrostatic stability of the aqueous dispersion. Due to this negative zeta potential, the diatomite is readily dispersible in distilled water, allowing the entire surface of the diatomite particles to be impregnated with iron(III) citrate solution, thus allowing the formed magnetic particles to be evenly distributed on the surface of the silica. The diatomite-supported catalyst was also tested, its zeta potential (−4 ± 5 mV) being shifted toward positive values compared to that of the pristine support (Figure 1B).

**Figure 2:**
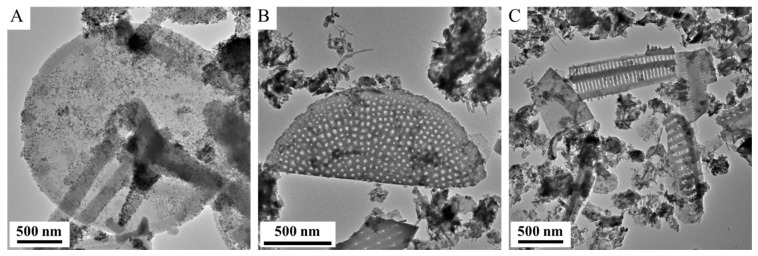
TEM pictures of diatomite particles with various sizes and morphologies (**A**–**C**).

TEM pictures of the magnetite-coated diatomite particles show a variety of morphologies and sizes (Figure 2).

**Figure 3:**
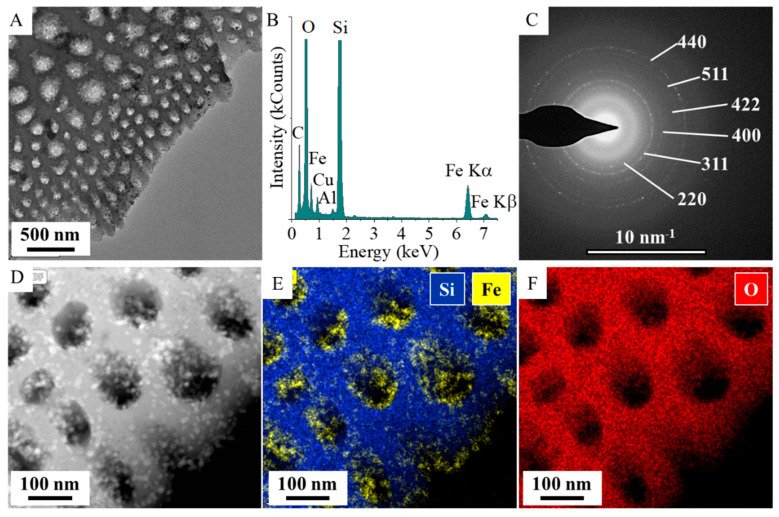
TEM picture (**A**), EDS (**B**), SAED (**C**), and elemental maps (**D**–**F**) of the magnetite-coated diatomite.

The TEM picture shows the surface of the support being richly covered by nanoparticles, which are concentrated mainly in the pores. This is also supported by the elemental maps (Figure 3A,D,E). On the energy-dispersive X-ray spectrum (EDS), in addition to the constituents of diatomite (oxygen, silicon, and aluminum), iron was also identified due to the presence of the magnetic nanoparticles (Figure 2B). Selected area electron diffraction (SAED) measurements confirmed that the diffraction pattern of the nanoparticles on the diatomite surface is consistent with the reflections typical of Fe_3_O_4_ crystallites (Figure 2C). The SAED results correlate well with the results of the XRD measurements.

**Figure 4:**
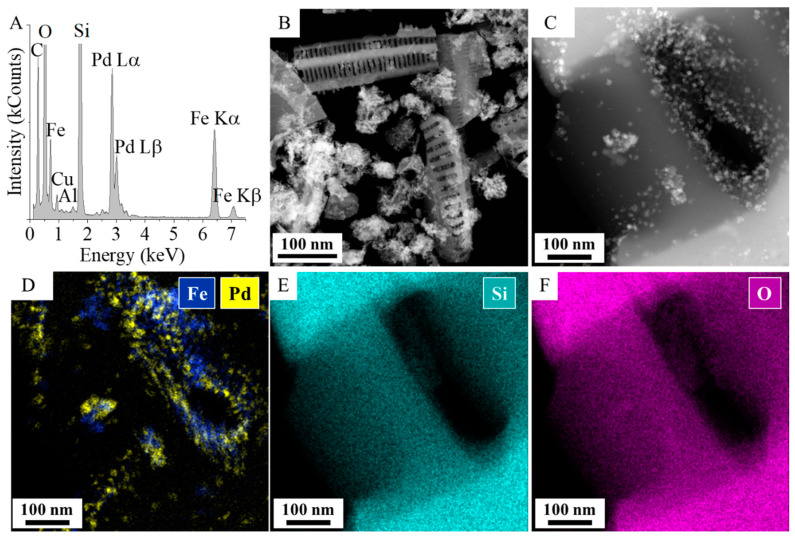
EDS (**A**), HAADF TEM picture (**B**,**C**), and elemental maps (**D**–**F**) of the palladium and magnetite-coated diatomite.

After deposition of palladium nanoparticles on the magnetite-coated diatomite-based support, EDS measurement was performed, confirming the presence of palladium (Figure 4A). TEM images taken in high-annular dark field mode show Pd and Fe_3_O_4_ nanoparticles with bright contrast (Figure 4B,C). Based on TEM pictures and elemental maps (Figure 4C,D), the formation of Pd crystal clusters took place at around the pores which are preferred in terms of binding energy. The elemental maps reveal that iron and palladium particles are located mainly together (Figure 4D). The close contact between Pd and Fe_3_O_4_ is advantageous in the field of catalysis, as electron transfer occurs between palladium and iron oxides with low oxidation states such as magnetite [33]. Magnetite transfers electrons to the originally electron-poor palladium, optimizing the electron distribution of the noble metal, which in turn enhances hydrogen bonding to the electron-rich Pd surface [34]. Moreover, Youmbi et al. used Density Functional Theory (DFT) in the gradient approximation to study of the charge transfer between magnetite substrate and palladium nanoparticles [35]. They found that the deposition of Pd nanoparticles on the magnetite surface led to charge transfer between the surface and the palladium atoms via donation-back-donation mechanism. Inside the palladium clusters on the magnetite surface, the density of positively charged Pd atoms become relatively low. Because the charges of the neighboring Pd atoms in the cluster become more negative, these negative charges could be attracted either by the topmost, positively charged Pd atoms or by the cations on the oxide surface. In this sense, the presence of more electrons at the interface will result in the reduction of surface Fe^3+^ ions, increasing the Fe^2+^/Fe^3+^ ratio only at the topmost surface layer. Similar results were obtained by Coker et al. when X-ray Magnetic Circular Dichroism (XMCD) spectroscopy measurements were applied for the confirmation of the reduction of Fe^3+^ ions [36]. The stoichiometric Fe^2+^/Fe^3+^ ratio in our magnetite support increased from 0.64 to 0.70 after deposition of palladium nanoparticles on the surface. The adsorption of palladium on Fe_3_O_4_ leads to metallic behavior by enhancement of the Density of States at the Fermi Level. 

An additional advantage of this electron transfer lies in the potential chemoselectivity that can be achieved during the hydrogenation process. Namely, the electron-rich palladium does not prefer aromatic rings that are also rich in electrons [37]. Owing to this, there will be no electrostatic attraction between them to allow the aromatic rings to adsorb horizontally on the catalyst surface. Instead, the reactant molecule, e.g., benzophenone, binds on the catalyst surface via the oxygen atom of its keto group to form the activated complex.

**Figure 5:**
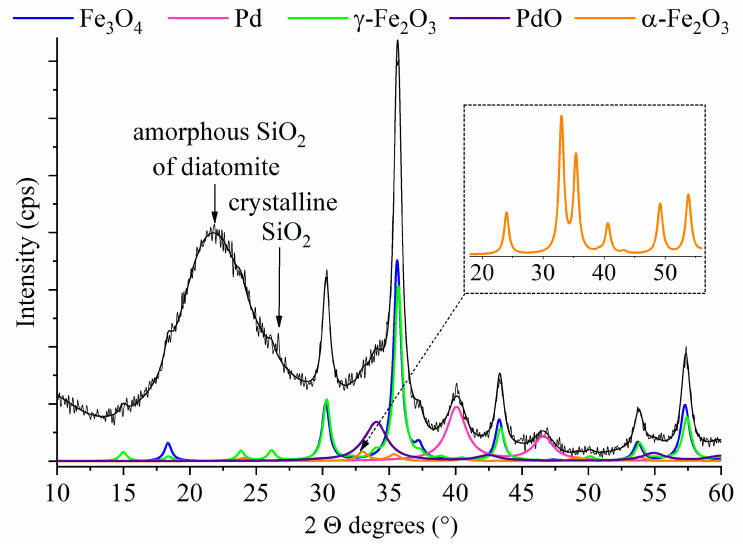
Rietveld refinement of XRD pattern of the Pd/Fe_3_O_4_–diatomite catalyst.

To identify the crystalline phases in the catalyst, XRD measurement was carried out (Figure 5). After Rietveld refinement of the X-ray diffractogram, reflections at 2θ values 18.3°, 30.2°, 35.6°, 43.3°, 53.7°, and 57.2° were found, which are characteristic to the (111), (220), (311), (400), (422), and (511) Miller-indexed Bragg peaks of the magnetite, respectively (PDF 19-629). However, in addition to magnetite, there is also a magnetic iron oxide phase, namely maghemite (γ-Fe_2_O_3_) in the sample with its characteristic reflections at 15.0° (110), 18.3° (111), 23.9° (210), 26.1° (211), 30.3° (220), 35.7° (311), 43.4° (400), 53.8° (422), and 57.4° (511) two theta degrees (PDF 39-1346). A small amount of hematite (α-Fe_2_O_3_, 2.23 wt%) also can be found on the diatomite surface with reflections at 33.1° and 35.4° two theta degrees (PDF 33-0664). The magnetite, maghemite, and hematite content of the catalyst are 20.9, 22.1, and 2.20 wt%, respectively. Figure 3 shows reflexions at 24.1° (101), 33.0° (210), 35.5° (110), 35.4° (311), 40.6° (511), 49.1° (702), and 53.9° (811) corresponding to the hematite phase, which is present in the catalyst only in a small amount (2.21 wt%) (PDF 33-0664). The (111) and (200) reflections of the elemental palladium are located at 40.1° and 46.5° two theta degrees, respectively. The Pd content of the catalyst is 4.40 wt% (PDF 46-1043). However, in addition to palladium, we also detected the presence of a palladium(II) oxide phase (3.31 wt%), with the corresponding (101), (110), and (112) reflections at 34.0°, 42.5°, and 54.9° two theta degrees, respectively (PDF 85-0713). Based on the results of the XRD analysis, it can be concluded that, in the prepared catalyst, the diatomite is in amorphous form with 47.1 wt%. A wide peak on the diffractogram in the area of approx. 15–24° two theta refers to amorphous SiO_2_ [38]. The composition is summarized in Table 1.

According to the CO_2_ adsorption measurements, the specific surface area of the catalyst is 35.6 m^2^/g.

### 2.2. Results of the Hydrogenation Reactions

**Figure 6:**
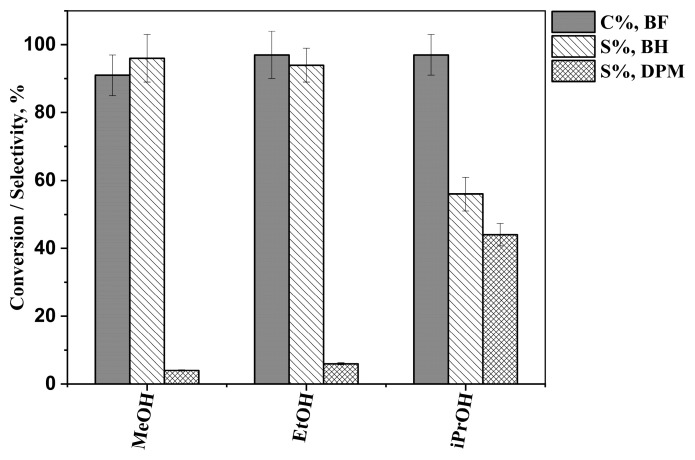
Benzophenone conversions (C%, BF), benzhydrol selectivities (S%, BH), and diphenylmethane selectivities (S%, DPM) in different solvents, after 80 min of reaction time.

**Figure 7:**
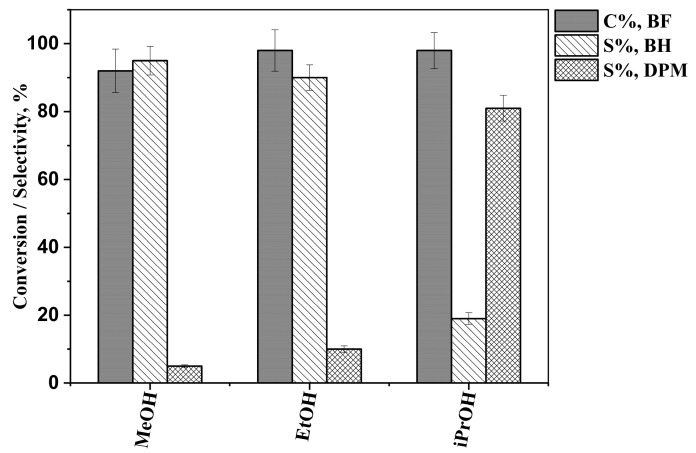
Benzophenone conversions (C%, BF), benzhydrol selectivities (S%, BH), and diphenylmethane selectivities (S%, DPM) in different solvents, after 240 min of reaction time.

Analysis of the reaction mixtures showed that Pd/Fe_3_O_4_–diatomite catalyzes the hydrogenation of benzophenone. Besides benzhydrol and/or diphenylmethane, no other products were detected by GC-MS. The outcome of hydrogenation was markedly different depending on the solvent. At 80 min of reaction time, using either methanol or ethanol resulted in high benzhydrol selectivity, 96 ± 7% and 94 ± 5%, respectively (Figure 6). However, in isopropanol, the benzhydrol selectivity was only 56 ± 5%. These differences were more pronounced when the reactions were allowed to run further in time. Namely, at 240 min of reaction time, in isopropanol, the benzhydrol selectivity decreased to 19 ± 2%, while in methanol and ethanol, it decreased only by 1–4 percent points (Figure 7). Benzophenone conversion at 80 min was 91 ± 6%, 97 ± 7%, and 97 ± 6% in methanol, ethanol, and isopropanol, respectively. At 240 min, 92 ± 6%, 98 ± 6%, and 98 ± 5% were achieved in methanol, ethanol, and isopropanol, respectively.

**Figure 8:**
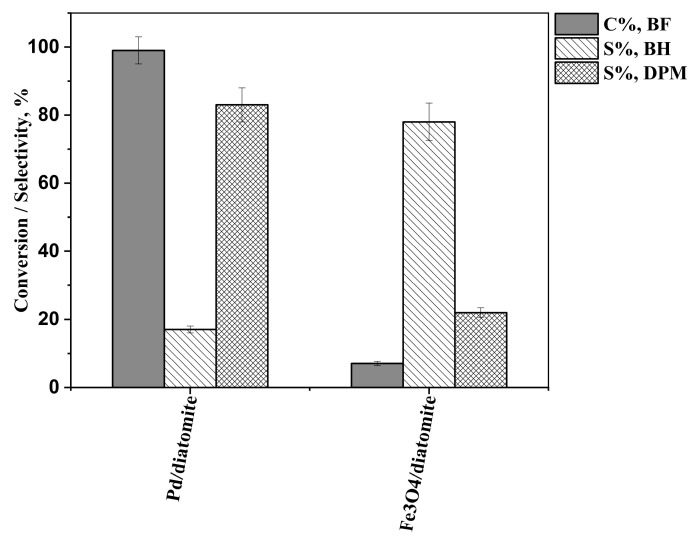
Benzophenone conversions (C%, BF), benzhydrol selectivities (S%, BH), and diphenylmethane selectivities (S%, DPM) in isopropanol, using only Pd or only Fe_3_O_4_ on the diatomite support, after 240 min of reaction time.

Since the prepared catalyst is a complex mixture, we tested the Fe_3_O_4_–diatomite support without Pd and also diatomite with only Pd on the surface to check which component is actually responsible for the catalytic effect and how they affect benzophenone conversion and product selectivity (Figure 8). The results provided by the Pd/diatomite catalyst are very similar to that of the Pd/Fe_3_O_4_–diatomite catalyst in isopropanol as the test solvent. It is quite evident that, in the absence of Fe_3_O_4_, the catalyst maintains its activity. However, without palladium, the Fe_3_O_4_–diatomite support showed only very low (approx. 8% conversion) activity. Although this low conversion makes the evaluation of the product selectivity less meaningful, the latter is the opposite of that observed with the Pd-containing catalyst. Regarding the active catalytic component, it can be concluded that the actual catalyst is the palladium. The Fe_3_O_4_ particles, however, provide an easy way to separate the catalyst from the medium.

## 3. Experimental

### 3.1. Materials

Palladium(II) nitrate dihydrate, Pd(NO_3_)_2_·2H_2_O (Alfa Aesar Ltd., Ward Hill, MA 01835, USA), and ethanol, C_2_H_5_OH (Lach-Ner, s.r.o., CS-27711 Neratovice, Czech Republic), were used to deposit Pd. For preparation of the magnetite nanoparticles onto surface of the diatomite support, iron(III) citrate, C_6_H_5_FeO_7_·xH_2_O (Acros Organics Ltd., B-2440 Geel, Belgium) was used as a precursor. Hydrogen (purity 4.0, Messer Group Ltd., 1044 Budapest, Hungary) and benzophenone, C_13_H_10_O (ThermoFisher GmbH, 76870 Kandel, Germany), were applied as reactants during the catalytic hydrogenation tests. Isopropanol (IPA), (CH_3_)_2_CHOH, methanol, CH_3_OH, and ethanol, C_2_H_5_OH, (all of them from VWR International S.A.S., F-93114 Rosny-sous-Bois-cedex, France) were used as solvents during of the hydrogenation tests. The diatomaceous earth was taken from an open-pit mine from Erdőbénye, Zemplén Mountain, Hungary.

### 3.2. Synthesis of the Magnetite-Coated Diatomite-Supported Palladium Catalyst

To the solution of 5.001 g iron(III) citrate in distilled water (200 mL), 5.002 g diatomaceous earth was added. An aqueous dispersion was prepared by ultrasonication for 5 min, after which the water was removed with in vacuo using a rotary evaporator. The remaining solid was dried at 105 °C overnight. The diatomite impregnated with iron citrate was heat-treated at 400 °C in a nitrogen atmosphere for two hours. Subsequently, a dispersion of 1.001 g magnetite-coated diatomite, a solution of 0.1282 g palladium(II) nitrate hydrate (39 wt% Pd content) in 10 mL distilled water and 100 mL ethanol was stirred for two hours at 50 °C. The solid phase was collected by magnetic separation, washed with distilled water, and dried overnight at 105 °C.

### 3.3. Characterization Technics

The diatomite-supported magnetic catalyst was examined by high-resolution transmission electron microscopy (HRTEM, Talos F200X G2, Thermo Scientific, Waltham, MA, USA) with field emission electron gun, X-FEG (accelerating voltage: 20–200 kV), for the characterization of the particle size and morphology. For imaging and electron diffraction, a SmartCam digital search camera (Ceta 16 Mpixel, 4k × 4k CMOS camera, Thermo Scientific, Waltham, MA, USA) and a high-angle annular dark-field (HAADF) detector were used. Sample preparation was carried out from the aqueous dispersion of the nanoparticles by dropping on 300 mesh copper grids (Ted Pella Inc., Redding, CA, USA). The identification and quantification of the crystalline phases were carried out by X-ray diffraction (XRD) measurements. Bruker D8 Advance diffractometer (Bruker, Karlsruhe, Germany, Cu-Kα source, 40 kV and 40 mA) in parallel beam geometry (Göbel mirror) with Vantec1 detector was applied. The average crystallite size was calculated by the mean column length-calibrated method by using full width at half maximum (FWHM) and the width of the Lorentzian component of the fitted profiles. Functional groups on the surface of the diatomite support were studied by using Fourier transform infrared spectroscopy (FTIR, Bruker Vertex 70 spectrometer, Bruker, Karlsruhe, Germany). The sample (10 mg) was pelletized with potassium bromide (250 mg) and the IR spectrum was obtained in transmission mode. The specific surface area of the catalyst was measured by CO_2_ adsorption–desorption experiments at 273 K by using a Micromeritics ASAP 2020 sorptometer (Micromeritics, Norcross, GE, USA) based on the Dubinin–Astakhov (DA) method. Gas chromatography coupled with mass spectrometry (GC-MS) is a suitable method for both qualitative and quantitative determination of the possible reaction products. The progress of hydrogenation was followed by a Shimadzu GCMS-QP2020 mass spectrometer-coupled gas chromatograph (Shimadzu, Duisburg, Germany). The reaction mixture contained 1-decanol (830 mg L^−1^) as an internal standard. Chromatographic separation was accomplished using a Stabilwax-MS capillary column (30 m length × 0.25 mm i.d., 0.25 µm film thickness) from Restek Corp. The column temperature program was as follows: 403 K (1 min), 403–523 K (10 K min^−1^), 523 K (2 min). The injector and the detector were set at 523 K and 503 K, respectively. Helium was used as carrier gas at 0.86 mL min^−1^ column flow rate. Electron ionization (EI) mode was performed with 70 eV electron energy. MS cut time was 3 min. 1 μL sample from the reaction mixture was injected by a Shimadzu AOC-6000 autosampler. The split was set at 1:300.

### 3.4. Catalytic Tests of the Catalyst in Benzophenone Hydrogenation

The hydrogenation of benzophenone was carried out in methanol, ethanol, and isopropanol (IPA). The concentration of benzophenone was 0.010 mol L^−1^. Approximately, 0.15 g catalyst was added to 150 mL benzophenone solution. A Büchi Uster Picoclave reactor system equipped with a 200 mL stainless steel vessel and a heating jacket was used for the hydrogenation tests. The pressure of H_2_ was kept at 20 bar, and the reaction mixture was thermostated at 323 K. Sampling was carried out after 5, 10, 15, 20, 30, 40, 60, 80, 120, 180, 200, 220, and 240 min.

The efficiency of the catalyst was characterized by calculating the conversion (*X*%) of benzophenone based on the following equation (Equation (1)):

(1) $$X\%=\frac{n_{consumed\;benzophenone}}{n_{initial\;benzophenone}}\;\cdot \;100$$

Diphenylmethane (DPM) yield (*Y*%) was also calculated as follows (Equation (2)):

(2) $$Y\%=\frac{n_{formed\;DPM}}{n_{theoritical\;DPM}}\;\cdot \;100$$

Furthermore, diphenymethane (DPM) selectivity (*S*%) was calculated according to the following equation (Equation (3)):

(3) $$S\%=\frac{n_{formed\;DPM}}{{\sum n}_{products}}\;\cdot \;100$$

## 4. Conclusions

Diatomaceous earth acts as an excellent catalyst support, providing high specific surface area and porosity with plenty of advantageous binding sites for both the iron oxide and palladium particles. Preparation of magnetite nanoparticles on the surface of the SiO_2_ leads to a significant simplification of the catalyst recovery process.

The catalytic results show that in the heterogeneous catalytic benzophenone reduction, the preferred product, i.e., benzhydrol or diphenylmethane, can be selected by changing the solvent. Using methanol or ethanol, the selectivity towards benzhydrol is higher; meanwhile, in isopropanol the main product is diphenylmethane. Regarding the complexity of the heterogeneous catalytic processes, there are usually no single or simple reasons (e.g., solvent polarity or hydrogen solubility in the solvent) by which the given chemoselectivity can be fully understood [39]. However, it is well known that the solvent plays a critical role in the outcome of a catalytic reaction [40]. In our case, isopropanol establishes a much higher rate of diphenylmethane formation than the other two alcohols, namely ethanol and methanol. The reason for this difference may be found either in a different solvent–product interaction or in a possible transfer hydrogenation process, the latter promoted by only isopropanol, giving rise to the hydrogenolysis of benzhydrol to diphenylmethane.

## Figures and Tables

**Table 1:** Composition of the Pd/Fe_3_O_4_–diatomite catalyst.

	Pd	PdO	Fe_3_O_4_	γ-Fe_3_O_4_	α-Fe_3_O_4_	SiO_2_
Content	4.40 wt%	3.31 wt%	20.9 wt%	22.1 wt%	2.23 wt%	47.1 wt%

## Data Availability

Data are contained within the article.
